# Similarity assessment of disulfide isoform profiles between the romosozumab biosimilar and the reference medicinal product

**DOI:** 10.1038/s41598-026-51578-9

**Published:** 2026-05-07

**Authors:** Dandan Zhao, Yanling Liu, Chun Wu, Chunlai Cao, Bohao Zhou, Yongchun Lin, Qiumei Liu, Guanheng Li, Jianrui Shi, Wenyu Chen, Yongjie Lai, Jing Li

**Affiliations:** 1 Zhuhai United Biopharma Co., Ltd, 399 Airport West Road, Zhuhai, Guangdong China; 2 Zhuhai United Laboratories Co., Ltd, 2428 Anji Road, Zhuhai, Guangdong China; 3 Department of Microbiology and Immunology, Zunyi Medical University (Zhuhai Campus), 368 Golden Coast Avenue, Zhuhai, Guangdong China

**Keywords:** Biosimilar, Disulfide isoform, Hydrophobicity, Bioactivity, Biochemistry, Drug discovery

## Abstract

Human IgG2 subclass antibodies exhibit complex interchain disulfide bonding patterns, resulting in three conformational structures: IgG2-A, IgG2-A/B and IgG2-B. The structural patterns of IgG2 disulfide isoforms fundamentally influence a spectrum of critical attributes, from molecular properties such as surface charge distribution, hydrophobicity, and N-glycosylation to ultimate biological activity and potency. Evenity (romosozumab) is a humanized IgG2 monoclonal antibody targeting sclerostin, for the treatment of osteoporosis. During the development of a romosozumab biosimilar, difference in the disulfide isoform profiles of the biosimilar and the reference medicinal product (RMP) was identified. Compared with RMP, the biosimilar presented a reduced IgG2-B content, accompanied by elevated IgG2-A/B and IgG2-A contents. The disulfide isoforms were fractionated via CEX-HPLC or enriched via a redox procedure, after which the key physicochemical properties, binding ability and potency were investigated. The analytical data revealed that, despite observed differences in specific physicochemical properties—including surface charge distribution, hydrophobicity, and high mannose content—the biosimilar’s disulfide isoforms exhibited comparable potency to the RMP. These findings suggest that the observed disulfide isoform profile differences do not compromise the potency of romosozumab under the conditions tested.

**Supplementary Information:**

The online version contains supplementary material available at 10.1038/s41598-026-51578-9.

## Introduction

The human IgG2 subclass has emerged as an attractive framework for therapeutic antibodies in clinical applications for which effector functions are undesirable or unnecessary for therapeutic activity^1–3^. IgG2 antibodies contain four disulfide bonds in the hinge region, resulting in multiple naturally occurring disulfide isoforms. These are known as IgG2-A, IgG2-A/B, and IgG2-B, classified according to the number of Fab arms that are linked via disulfide bonds to the heavy chain hinge region^4,5^. The IgG2-A and IgG2-B isoforms have symmetrical interchain disulfide connectivity in that the same light-heavy chain (LC-HC) attachment is present on both sides of the molecule. In the third isoform (IgG2-A/B), one light chain was connected to CH1, whereas the other was connected to the hinge region, resulting in an asymmetrical structure. The IgG2-A isoform is depicted as a canonical Y-shaped structure, and the IgG2-B isoform is depicted as a constrained T-shaped structure^4–6^.

Secretory cells initially produce primarily IgG2-A, which is rapidly converted to IgG2-A/B, followed by slow conversion to IgG2-B while circulating in the blood^6,7^. The IgG2-A/B disulfide isoform is considered an intermediate between IgG2-A and IgG2-B. The variation in conformational structure, arising from interchain disulfide bond connectivity patterns, has the capacity to impact key physicochemical characteristics and the potency of antibodies. Far-UV CD analysis demonstrated that all the IgG2 disulfide isoforms and the original material presented no significant differences in secondary structure^5^. In the SEC‒HPLC analysis, IgG2-A was eluted earlier than IgG2-B, indicating its larger apparent molecular size. In sedimentation velocity analytical ultracentrifugation, the enriched IgG2-B isoform consistently presented a higher sedimentation coefficient (S) value than did the IgG2-A isoform. These results indicated that the IgG2-B isoform has a more compact structure^5^. The IgG2-A and original IgG2 control samples produced similar thermograms in the DSC assay, whereas the IgG2-B sample showed a significantly higher enthalpy during the high-temperature transition^5^. Given the different inter-chain disulfide bond connectivity patterns, it is reasonable to expect that IgG2 disulfide isoforms display altered tertiary and quaternary structures.

N-glycosylation is a complex process in which a dolichol pyrophosphate donor transfers a tetradecasaccharide precursor en bloc to the nitrogen atom of the asparagine side chain within the consensus sequence N–X–S/T, where X can be any amino acid except proline^8^. This process initiates in the endoplasmic reticulum (ER), where premature N-glycans undergo trimming and remodeling during transport to the Golgi apparatus, culminating in the formation of mature N-glycans in the Golgi^9^. Covalent disulfide bonds are initially assembled in the ER, disulfide bond shuffling may occur later along the secretory pathway and in the extracellular microenvironment^10^. Multiple studies have indicated a relationship between N-glycan maturation and disulfide bond formation. Depending on the protein sequence and conformational structure, these two post-translational modification processes may act independently, cooperatively, or in a mutually antagonistic manner^11^. Given that disulfide isoform formation occurs prior to N-glycan maturation and that IgG2 exists as three disulfide isoforms, the distinct conformational structures of these isoforms may affect the later stages of N-glycan processing, particularly in the Golgi apparatus. A study has indicated that IgG2 disulfide isoforms exhibit differences in their N-glycan structure^7^. The IgG2 mAb was purified from the lysed CHO cells and the supernatant. RP-HPLC analysis revealed that the unsecreted IgG2s were predominantly IgG2-A and that the main types of glycans were high-mannose forms of Man8 and Man9. However, the predominant forms of secreted IgG2 were IgG2-A/B and IgG2-B, and the main types of glycans were G0F and G1F^7^. Removal of N-linked glycans by PNGase F digestion did not alter the disulfide isoform profiles. These data suggest that the structural distinction between these isoforms is primarily independent of glycosylation^12^. On the other hand, the structural variation brought about by disulfide isoforms might have an impact on N-glycan trimming and maturation in antibody producing cells.

Notably, determining whether disulfide isoforms, which have been demonstrated to engender discrepancies in surface charge distribution, hydrophobicity, conformational structure, hydrodynamic radius, and the N-glycosylation profile, have the capacity to influence the potency of an antibody is of critical importance. Two independent studies on this issue have yielded seemingly contradictory yet independently valid findings. In one study, the relative potency of an IgG2 subclass anti-EGFR antibody and its IgG2-B disulfide isoform was assessed via both ELISA binding and a cell-based reporter gene assay. These assays revealed that the two molecules exhibited comparable potencies^12^. Another study established that the original anti-IL-1R1 IgG2 subclass mAb and enriched IgG2-A and IgG2-B exhibited divergent levels of potency with regard to the inhibition of IL-1β-induced IL-6 production in human chondrocytes and human whole blood. The potency of IgG2-A was significantly greater than that of the original IgG2 sample, which in turn was more potent than that of IgG2-B^5^.

The disulfide isoform profile of an IgG2 mAb biosimilar may diverge from that of its RMP. The disulfide isoform profile should be included as a critical quality attribute (CQA). Currently, there is no standard guideline to instruct how to assess the similarity of this CQA. Furthermore, published studies that have systematically analyzed the disulfide isoform profiles of IgG2 subclass biosimilars are lacking. In one study, RP-HPLC analysis of two proposed denosumab biosimilars and RMP revealed similar disulfide isoform patterns and contents relative to those of the EU/US reference products (Prolia/Xgeva). However, the authors also noted a quality drift in the RMP, characterized by an increase in IgG2-B and a decrease in IgG2-A/B and IgG2-A1^13^. Consequently, the profiles of the biosimilars differed from those of the post-drift RMP batches. This study did not elucidate whether the observed isoform difference affected critical physicochemical properties or biological potency^13^. In the development of a romosozumab biosimilar (an anti-sclerostin IgG2 mAb for osteoporosis), assessment revealed a dissimilarity in the disulfide isoform profile compared with that of the reference product. The biosimilar displayed a relatively low IgG2-B level alongside elevated levels of IgG2-A/B and IgG2-A. To determine the key physicochemical properties and potency of these isoforms, they were isolated via either redox enrichment or CEX-HPLC fractionation. The collected isoforms then underwent systematic analysis alongside the biosimilar and the RMP.

## Results

### The biosimilar exhibited a disulfide isoform profile different from that of the RMP

**Fig. 1:**
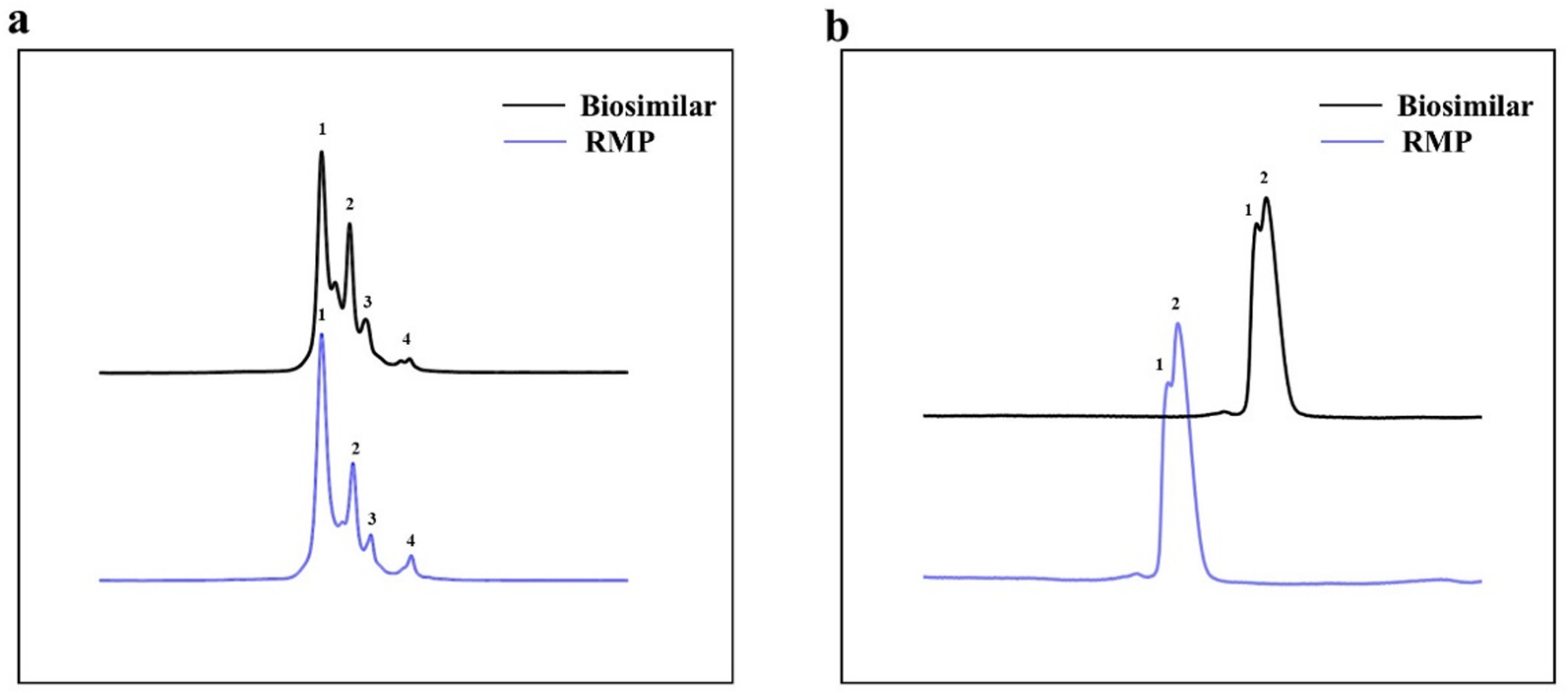
Analysis of the romosozumab biosimilar and RMP by RP-HPLC (**a**) and nrCE-SDS (**b**). In panel a, peak 1 is assigned to IgG2-B, peak 2 to IgG2-A/B, and peaks 3 and 4 to IgG2-A. In panel b, the migration times of RMP and the biosimilar were adjusted to facilitate direct comparison of peak profiles.

RP-HPLC is widely regarded as the most optimal method for the analysis of disulfide isoforms of IgG2^14–16^. The disulfide isoforms interact differently with the column matrix and are eluted sequentially, resulting in heterogeneous profiles. As demonstrated in Fig. 1a, the biosimilar (black line) and RMP (blue line) exhibited four peaks. Based on previous reports^4,5,15^, peak 1 is assigned to IgG2-B, peak 2 to IgG2-A/B, and peaks 3 and 4 to IgG2-A. The biosimilar presents a comparatively low level of IgG2-B but a relatively high level of IgG2-A/B and IgG2-A. IgG2-B has the shortest retention time on the column, presumably due to its relatively compact structure and less exposed hydrophobic surface area than those of IgG2-A/B and IgG2-A. IgG2-A/B was eluted immediately after IgG2-B, followed by IgG2-A, which has the most open structure. The disulfide isoform distributions differ between the biosimilar and RMP. The biosimilar consists of 55.76% IgG2-B, 25.03% IgG2-A/B, and 19.21% IgG2-A, whereas the RMP contains 62.12%, 20.22%, and 17.66% of these isoforms, respectively.

The other technique for analyzing disulfide isoforms is nrCE-SDS^4,12^. As demonstrated in Fig. 1b, the biosimilar and RMP both exhibited a double peak, which was denoted as structural isoforms 1 and 2. The relative percentages of the structural isoforms in the biosimilar were approximately 32.57% for isoform 1 and 65.95% for isoform 2. The corresponding percentages of the two structural isoforms for RMP were 26.83% and 71.64%, respectively. In nrCE-SDS, proteins coated with SDS are separated based on their molecular mass. The disulfide isoforms share an identical amino acid sequence, which rules out primary sequence as the cause of the difference in hydrodynamic radius. Consequently, the data indicate that the variation can be attributed to conformational differences arising from divergent disulfide bond connectivity.

## The disulfide isoform profiles were relatively stable throughout the stability studies

**Fig. 2:**
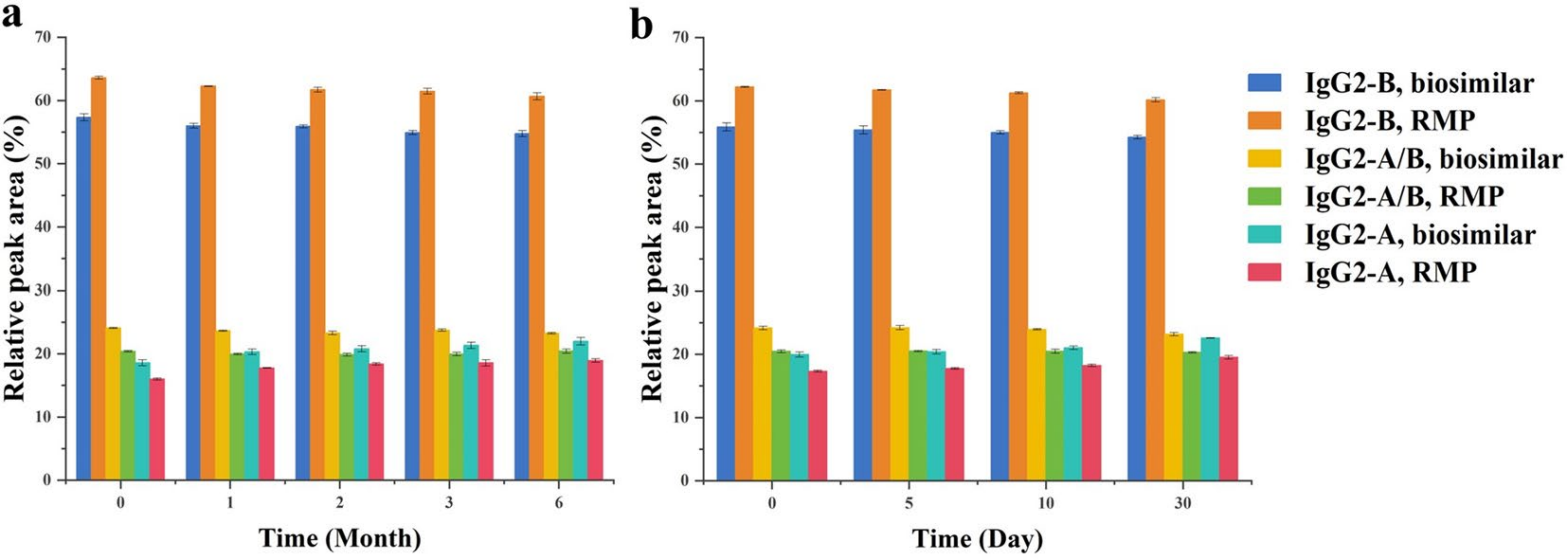
Relative percentages of disulfide isoforms of the biosimilar and RMP in accelerated and stress stability studies. (**a**). The biosimilar and RMP were incubated at 25 °C for 6 months, and the samples collected at 0, 1, 2, 3, and 6 months were analyzed via RP-HPLC. (**b**). The biosimilar and RMP were incubated at 40 °C for 30 days, and the samples collected at 0, 5, 10, and 30 days were analyzed via RP-HPLC. The relative percentages of the disulfide isoforms were calculated and plotted on bar graphs. Data were obtained from three batches of the biosimilar and three batches of the RMP.

In vivo studies suggest quick conversion of IgG2-A to IgG2-A/B, followed by slow conversion to IgG2-B, in the human circulation^6,7^. It is crucial to ascertain whether the disulfide isoform profiles undergo similar alterations during storage. We investigated the stability of disulfide isoform profiles through accelerated, stress, and long-term studies. In the accelerated stability study (25 °C), both the biosimilar and RMP were sampled over 6 months and analyzed via RP-HPLC (Fig. 2a). The relative levels of disulfide isoforms remained consistent throughout this period. Similarly, under stress condition (40 °C for 30 days), RP-HPLC analysis confirmed that the disulfide isoform profiles of the biosimilar and RMP were relatively stable (Fig. 2b). Finally, in the long-term study (2–8 °C for up to 24 months) of the biosimilar, the relative content of disulfide isoforms were consistent (data not shown). Collectively, these results indicate that the disulfide isoform profiles of both the biosimilar and RMP remain consistent under long-term, accelerated, and stress stability studies.

## Enrichment of IgG2 disulfide isoforms with redox treatment

Demonstrating comparability in the key physicochemical properties, bioactivity, and potency of disulfide isoforms constitutes a vital step in the similarity assessment of biosimilar candidates. The disulfide isoforms can be transformed and enriched via a redox procedure. The redox procedure has previously been demonstrated to enrich for two of the IgG2 isoforms (IgG2-A and IgG2-B) when performed with or without GuHCl^5^. The IgG2-A or IgG2-B disulfide isoforms from the biosimilar and RMP were enriched via the redox procedure.

**Fig. 3:**
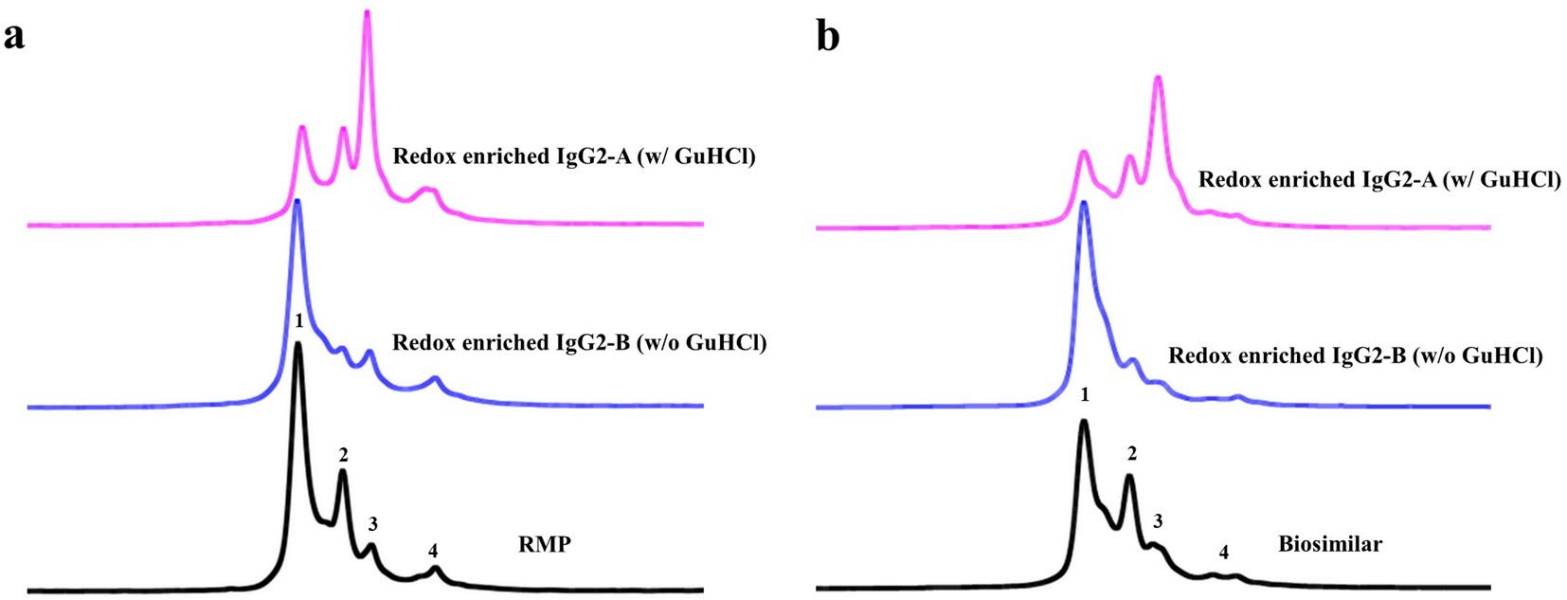
RP-HPLC analysis of the redox-enriched disulfide isoforms from RMP (**a**) and the biosimilar (**b**) in conjunction with the original samples.

As demonstrated in Fig. 3, the redox-enriched disulfide isoforms were analyzed via RP-HPLC. The presence of 1 M GuHCl resulted in the conversion of IgG2 disulfide isoforms toward IgG2-A (peaks 3 and 4); conversely, the absence of 1 M GuHCl led to the transformation of the disulfide isoforms toward IgG2-B (peak 1). The major RP-HPLC peaks in the redox-treated materials eluted at the same retention times as the corresponding peaks in the original control material that was not subjected to redox treatment. GuHCl is a denaturant; however, at a concentration of 1 M, it has been shown to have no effect on the overall secondary or tertiary structure of antibodies^17,18^. Therefore, the observed alterations in the RP-HPLC profile of the enriched IgG2-A are attributable to disulfide bond rearrangement rather than structural disruption induced by GuHCl.

## Fractionation of disulfide isoforms with low-pH CEX-HPLC

The distinct conformational structures of the IgG2 disulfide isoforms result in different surface charge distributions, leading to a complex elution profile in ion-exchange chromatography^4,19^. For this reason, the disulfide isoforms of IgG2 antibodies can be resolved via CEX-HPLC at a relatively low pH of 5.0^4^. In this study, the disulfide isoforms were fractionated via semipreparative CEX-HPLC at pH 4.9 ± 0.05.

**Fig. 4:**
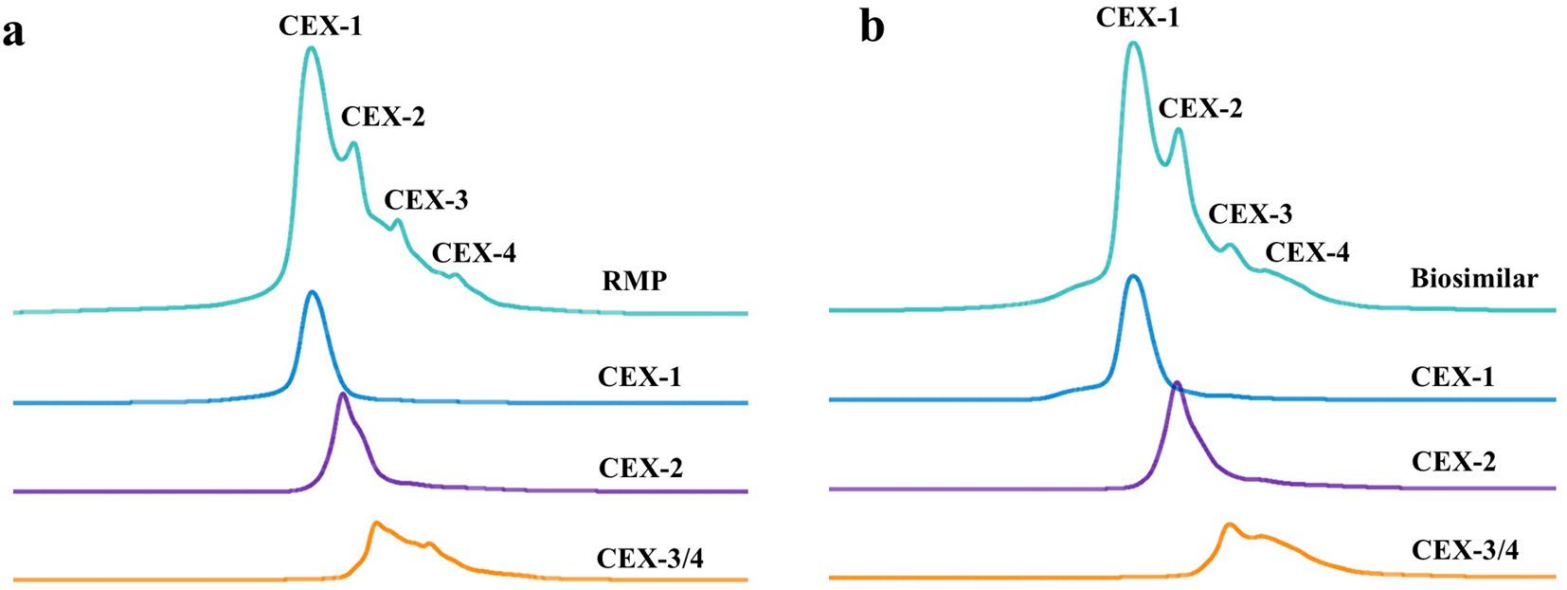
CEX-HPLC analysis of the fractionated disulfide isoforms from the biosimilar (**a**) and RMP (**b**) in conjunction with the original samples.

The collected fractions, along with the original biosimilar and RMP samples, were reanalyzed by low-pH CEX-HPLC. As shown in Fig. 4, the resulting peak shapes were analogous to those obtained by RP-HPLC. The biosimilar and RMP exhibited a total of four distinct peaks, labeled CEX-1, CEX-2, CEX-3 and CEX-4, according to the sequence of elution order. CEX-1 and CEX-2 were fractionated individually from the semipreparative column; owing to the relatively low quantities of CEX-3 and CEX-4, these two peaks were fractionated together and designated as CEX-3/4. The fractionated CEX-HPLC peaks were eluted at the same retention times as the corresponding peaks in the original RMP and biosimilar control materials. Despite the low-pH CEX-HPLC and RP-HPLC methods displaying analogous peak shapes for the biosimilar and RMP, it is crucial to acknowledge the fundamental distinctions between these two techniques. The CEX-HPLC fractions, designated CEX-1, CEX-2, and CEX-3/4, were not identical to the IgG2-B, IgG2-A/B, and IgG2-A fractions resolved via RP-HPLC. The purity of these fractions from the biosimilar was analyzed via RP-HPLC, and the majority of the CEX-1 and CEX-2 contents could be classified as IgG2-B and IgG2-A/B, respectively. For CEX-3/4, this fraction is composed of a mixture of IgG2-B, IgG2-A/B and IgG2-A, with percentages of 41.30%, 18.92%, and 39.77%, respectively (supplementary Fig. 1).

## CEX-HPLC-fractionated disulfide isoforms exhibit varying contents of high-mannose

**Fig. 5:**
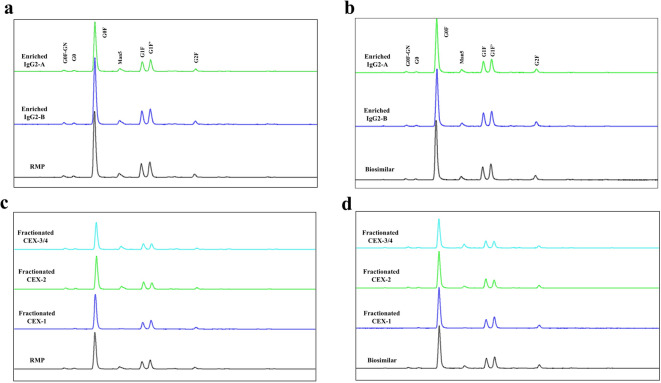
N-glycan profiles of the enriched isoforms and the fractionated isoforms. (**a**). Redox-enriched disulfide isoforms and the original RMP sample; (**b**). Redox-enriched disulfide isoforms and the original biosimilar sample; (**c**). Low-pH CEX-HPLC-fractionated disulfide isoforms and the original RMP sample; (**d**). The low-pH CEX-HPLC-fractionated disulfide isoforms and the original biosimilar sample.

To investigate whether disulfide isoforms exhibit divergent N-glycan profiles, the enriched isoforms and the fractionated isoforms in conjunction with their original control material were analyzed via hydrophilic interaction liquid chromatography (HILIC). As shown in Fig. 5, both RMP and the biosimilar, as well as the redox-enriched isoforms IgG2-A and IgG2-B, exhibited similar profiles for key glycosylation attributes, including galactosylation, fucosylation, high mannose, and sialylation. The three most prevalent N-glycans are G0F, G1F, and G1F’ (Fig. 5a, b; Table 1). Given that the enriched IgG2-A and IgG2-B were derived from the same original material (either the romosozumab biosimilar or the RMP), their similar N-glycan profiles are to be expected.

**Table 1:** Comparative analysis of glycosylation (galactosylation, fucosylation, high-mannose, sialylation) in the RMP, biosimilar, and redox-enriched isoforms.

Sample	Galactosylation (%)	Fucosylation (%)	High mannose (%)	Sialylation (%)
RMP	18.39	98.25	6.55	1.26
Enriched IgG2-A from RMP	18.42	98.23	6.46	1.30
Enriched IgG2-B from RMP	18.38	98.22	6.44	1.27
Biosimilar	21.21	98.24	5.68	1.17
Enriched IgG2-A from biosimilar	20.02	98.06	5.49	1.19
Enriched IgG2-B from biosimilar	21.67	98.21	5.54	1.26

The fractionated disulfide isoforms and their original material (biosimilar and RMP) demonstrated comparable levels of galactosylation, fucosylation and sialylation, with the exception of high mannose. As demonstrated in Fig. 5 (panels c and d) and Table 2, fractionated CEX-3/4 (a mixture of IgG2-A, IgG2-A/B and IgG2-B) presented higher levels of high mannose than did CEX-2 (mostly IgG2-A/B) and CEX-1 (mostly IgG2-B), with 11.36% vs. 8.62% and 2.12% for RMP and 8.85% vs. 4.65% and 1.29% for the biosimilar. Despite the variation in high-mannose content across disulfide isoforms, the overall levels remained comparable between the biosimilar (4.91%) and RMP (6.56%).

**Table 2:** Comparative analysis of glycosylation (galactosylation, fucosylation, high-mannose, and sialylation) in the RMP, biosimilar, and fractionated isoforms.

Sample	Galactosylation (%)	Fucosylation (%)	High mannose (%)	Sialylation (%)
RMP	18.40	98.23	6.56	1.28
Fractionated CEX-1 from RMP	19.03	98.49	2.12	0.69
Fractionated CEX-2 from RMP	17.80	97.72	8.62	1.59
Fractionated CEX-3/4 from RMP	17.79	97.90	11.36	1.38
Biosimilar	21.34	98.44	4.91	1.14
Fractionated CEX-1 from biosimilar	21.87	99.03	1.29	0.93
Fractionated CEX-2 from biosimilar	20.59	98.18	4.65	1.12
Fractionated CEX-3/4 from biosimilar	21.10	97.85	8.85	1.45

### IgG2 disulfide isoforms exhibit similar potency to RMP

**Fig. 6:**
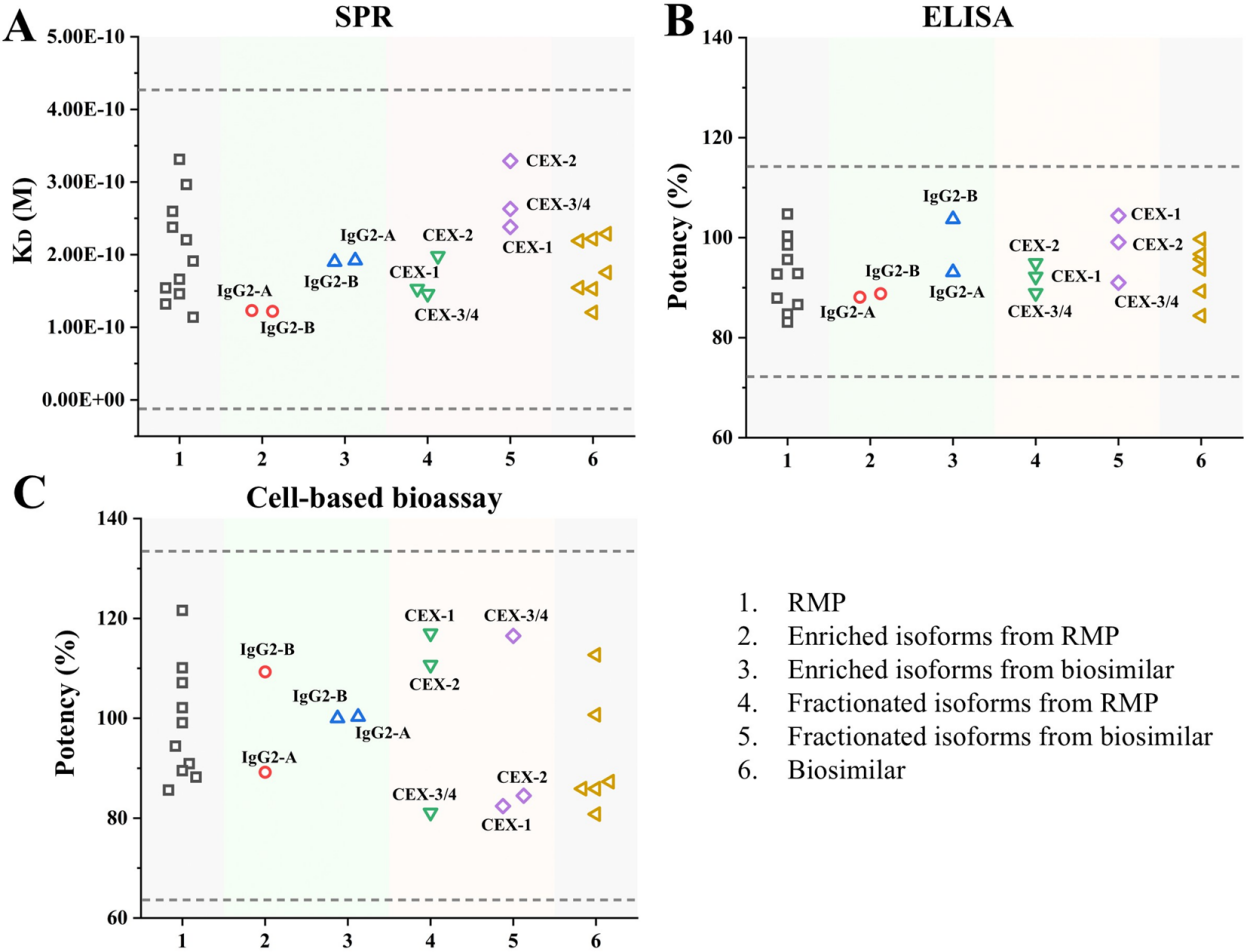
Comparison of the potency of enriched isoforms and fractionated isoforms with 10 batches of RMP and 6 batches of biosimilar. (**a**). Sclerostin binding affinity (SPR); (**b**). Sclerostin binding affinity (ELISA); (**c**). Bioactivity to reactivate the Wnt signaling pathway in a cell-based reporter gene assay. Each marker shows the activity value of a specific batch of the RMP and the biosimilar or the enriched isoforms and the fractionated isoforms. The dashed lines represent the quality range according to the data from multiple batches of RMP (mean ± 3 SD).

Romosozumab increases bone formation by specifically neutralizing sclerostin, a negative regulator that inhibits the Wnt signaling pathway in osteoblasts. Therefore, we investigated the binding affinity of these disulfide isoforms for sclerostin and their efficacy in reactivating Wnt signaling. The binding affinity (by SPR/ELISA) and potency (by cell-based assay) of the disulfide isoforms were evaluated. The analyses included redox-enriched material, CEX-HPLC fractions, and 10 batches of RMP alongside 6 batches of the biosimilar (Fig. 6a, b). All data originating from these disulfide isoforms were within the mean ± 3SD range of the RMP. These data demonstrate that the enriched isoforms and the fractionated isoforms not only exhibit binding ability for sclerostin similar to that of RMP, but also show comparable binding among themselves. The potency of the enriched isoforms and the fractionated isoforms in activating Wnt signaling was assessed via a cell-based reporter gene assay. As shown in Fig. 6c, all data points fell within the mean ± 3SD range of the RMP, indicating similar potency both to the RMP and across the various isoform preparations.

## Discussion

The romosozumab biosimilar exhibited a different disulfide isoform profile from RMP, characterized by a reduced level of IgG2-B alongside increased levels of IgG2-A and IgG2-A/B. The presence of different disulfide isoforms is an inherent property of the IgG2 subclass antibodies. The disparities in structure among these isoforms are attributed to the varied interchain disulfide bond linkage patterns rather than other posttranslational modifications^12^. Furthermore, the IgG2 disulfide isoforms have been shown to sequentially convert from IgG2-A to IgG2-A/B and finally to IgG2-B in human circulation^6,7^. The relative content of the IgG2 disulfide isoforms remained relatively constant throughout the stability studies, including accelerated stability, forced degradation and long-term stability studies. Given the formulation composition (acetate, calcium, polysorbate 20, sucrose)^20^, which is inert with respect to disulfide rearrangement, the isoform profile differences between the biosimilar and the RMP are established during manufacturing and remain unchanged prior to in vivo administration. Romosozumab is administered subcutaneously at a dosage of 210 mg once a month^21^. The half-life of romosozumab in humans is approximately 12.8 days. This pharmacokinetic profile supports the view that any difference in the disulfide isoform profile between the biosimilar and RMP would persist throughout the treatment cycle, as both products are expected to undergo similar dynamic transitions in the circulation. This raises the question of whether the difference in the disulfide isoform profile affects the potency of the biosimilar. The overall in vitro comparative studies in this study suggest the following: (1) The biosimilar and RMP exhibit similar binding abilities to sclerostin and equivalent potency in restoring the Wnt-1 signaling pathway; (2) The enriched isoforms and the fractionated isoforms from the biosimilar and RMP demonstrate similar binding ability and potency, despite the presence of varying levels of high mannose among the fractionated isoforms. Furthermore, a preclinical in vivo pharmacodynamic study indicated that the biosimilar and RMP have comparable efficacy in alleviating osteoporosis in ovariectomized rats (data not shown). These data suggest that the difference in the disulfide isoform profile is not expected to affect the potency of romosozumab biosimilar.

The process of disulfide formation within polypeptides commences during the cotranslational translocation of proteins into the ER^22^. Correct disulfides are introduced into proteins within the ER during folding and assembly^23^. The intra-ER microenvironment is characterized by elevated oxidation levels, rendering IgG2 disulfide isoforms susceptible to forming the IgG2-A subtype^24^. During the translocation of proteins from the ER to the Golgi apparatus and their subsequent secretion into extracellular fluids, the microenvironment changes, with a decrease in oxidation and an increase in reduction. This facilitates the conversion of IgG2-A to IgG2-A/B and IgG2-B. This aligns with reports that IgG2 from disrupted CHO cells is enriched in IgG2-A, whereas IgG2 from the cell culture supernatant is enriched in IgG2-A/B and IgG2-B^7^.

The process of N-glycosylation is initiated by the transfer of a tetradecasaccharide precursor en bloc from a dolichol pyrophosphate donor to the nitrogen atom of an asparagine side chain within a consensus sequence, such as N297 on the IgG heavy chain. The dolichol moiety was trimmed to a high-mannose-type glycan before the glycoproteins were dislocated to the Golgi apparatus. It is generally accepted that glycan heterogeneity is primarily established during cell culture, as glycosylation is governed by the availability and activity of glycosyltransferases and nucleotide sugars, both of which are influenced by cell culture process parameters such as culture medium, temperature, nutrient feed, dissolved oxygen, pH, and osmolality^25–27^.

Both N-glycosylation and disulfide bonds form posttranslational modifications on proteins in the ER while they pass through the secretory pathway^11,28^. Golgi mannosidases and multiple glycosyltransferases catalyze the processing of high-mannose glycans into hybrid or complex N-glycans. Processing and branching are modulated by glycan location: certain N-glycans are more accessible to Golgi glycosylation enzymes and are therefore more likely to be processed into complex N-glycans. Therefore, the N-glycosylation of a protein is not dictated solely by the canonical Asn-X-Ser/Thr sequon, glycosylation enzymes, and nucleotide sugars; the three-dimensional structure and local flexibility around the sequon are also critical determinants. Consequently, within a single protein, some glycosylation sites may be occupied by complex N-glycans, while others carry high-mannose or hybrid structures^29,30^. The results of our study demonstrated that CEX-HPLC-fractionated CEX-3/4 (a mixture of IgG2-A, IgG2-A/B and IgG2-B) and CEX-2 (mostly IgG2-A/B) have higher levels of high-mannose glycan, mostly Man5, than does CEX-1 (mostly IgG2-B). As IgG2 antibodies translocate from the ER to the Golgi apparatus, the intraorganellar environment becomes more reducing, which leads to the conversion of IgG2-A to IgG2-A/B and IgG2-B. At the same time, the N-glycan undergoes further trimming to form a complex glycan structure, with glycosyltransferases specifically located in the Golgi apparatus. Theoretically, conformational differences among IgG2-A, IgG2-A/B, and IgG2-B isoforms can influence accessibility to glycosylation enzymes, thereby promoting different N-glycan profiles. In contrast to those of IgG2-A, we propose that the conformational structures of IgG2-A/B and IgG2-B render them more accessible to mannosidases and glycosyltransferases, thereby promoting the formation of complex, branched, mature N-glycans.

The generation of disulfide isoforms takes place prior to the trimming of mannose and the branching of N-glycans. The disulfide isoforms could affect the trimming and maturation of N-glycans. In contrast, N-glycans do not influence the structural isoforms of disulfide^12^. The disulfide bonds of the aglycosylated antibody can be effectively and accurately assembled in CHO cells without the occurrence of N-glycosylation^31^. The CEX-HPLC-fractionated disulfide isoforms presented different levels of high-mannose content. A high mannose content was shown to significantly impact PK by decreasing the antibody half-life^32,33^. The overall high-mannose contents of the biosimilar and RMP are similar, and this subtle difference is not expected to result in any clinically meaningful impact on the PK of the biosimilar. In addition, in vivo preclinical PK analysis indicated that the biosimilar and RMP possess analogous PK properties (data not shown).

The natural human IgG subclasses have a unique hinge length and disulfide pattern. They are arranged in order of flexibility, from the most flexible to the least flexible: IgG3 > IgG1 > IgG4 > IgG2^34^. IgG2 is the major isotype produced against bacterial carbohydrates in response to infection^35^. IgG2 has a more rigid hinge region, which reduces flexibility but enhances stability. This makes it favorable for recognizing repetitive carbohydrate antigens, such as those found on bacterial capsules. Among IgG2 antibodies, IgG2-A is more flexible than IgG2-B, while IgG2-A/B exhibits intermediate flexibility. The disulfide bond structure of IgG2 acts as a covalent “switch” that controls antibody conformation and flexibility. These isoforms have been shown to affect the biological activity of an IL-1R1-blocking antibody^5^. In the case of receptor agonistic antibodies, the IgG2 disulfide isoforms behave differently. For CD40-targeting mAbs, the two isoforms exhibit starkly contrasting immunostimulatory activities: IgG2-A is inert and devoid of stimulatory activity, whereas IgG2-B is a potent agonist^36–38^. Furthermore, the principles of increasing agonism by restricting antibody conformation through disulfide modification can be translated to other receptors, such as CD200R and h4-1BB^39,40^. Sclerostin is a 24 kDa and 213 amino acid-long, positively charged glycoprotein with a secretion signaling peptide comprising the first 23 amino acids^41^. Given that antibody binding to sclerostin does not correlate with receptor activation and that sclerostin is relatively small compared with the antibody, the flexibility of the IgG2 antibody and its disulfide isoform profile are unlikely to affect overall potency.

Although the disulfide isoform profile of the romosozumab biosimilar differs from that of RMP, systematic analysis of the enriched isoforms and the fractionated isoforms revealed no consequential impact on bioactivity or potency. Given the unique properties of sclerostin and the mechanism of romosozumab, the conclusion of this study cannot be directly extrapolated to other IgG2 biosimilars. However, for the development of IgG2 biosimilars—particularly those that target receptors via avidity-driven binding—a comprehensive similarity assessment is recommended. This assessment should systematically evaluate disulfide isoform profiles and their impact on key physicochemical and biological properties, with a focused analysis of potency.

## Materials and methods

### Materials

RMP-romosozumab (Evenity^®^, 105 mg/1.17 mL) was purchased from Amgen Inc. Romosozumab biosimilar (105 mg/1.17 mL) was developed in house by Zhuhai United Biopharma Co., Ltd. (Zhuhai, Guangdong, China). Slide-A-Lyzer™ dialysis cassettes with a molecular weight cutoff (MWCO) of 10 K (10,000 Da) and cysteamine dihydrochloride were purchased from Pall Corporation. L-Cysteine was obtained from Sigma‒Aldrich. Guanidine-HCl was purchased from MP Biomedicals, LLC. Acetonitrile and isopropanol were purchased from Shanghai Xingke High-Purity Solvents Co., Ltd. and Tianjin Safery Co., Ltd., respectively.

### Reversed-phase high-performance liquid chromatography (RP-HPLC)

RP-HPLC analysis was carried out on an Agilent 1290 Infinity II system (Agilent Technologies, Santa Clara, CA, USA). The separation of the IgG2 disulfide isomers was achieved by employing an analytical HALO C4 column (1000 Å, 2.7 μm, 2.1 mm ID × 50 mm). Mobile phase A was composed of 10% acetonitrile and 2% isopropanol in an aqueous solution with 0.1% TFA. The composition of mobile phase B was 20% acetonitrile and 70% isopropanol in an aqueous solution with 0.1% TFA. The column was equilibrated with 22.6% B for five minutes, after which the IgG2 disulfide isomers were eluted by a linear gradient of mobile phase B from 22.6% to 28.6% over a period of 32 min. The flow rate was set at 0.2 mL/min, and UV absorption at 280 nm was used for detection. The column temperature was set at 85 °C, and the injection volume was 2 µl at a protein concentration of 2 mg/mL.

### Nonreduced capillary electrophoresis-sodium dodecyl sulfate (nrCE-SDS)

nrCE-SDS was performed via a CESI8000 Plus instrument (SCIEX, Framingham, MA, USA). Denatured samples were injected into a bare fused silica capillary and separated according to their hydrodynamic size, which was produced by the applied electric field. The migration time of small proteins is therefore inversely proportional to their overall size. Electrophoresis was performed, and the signals were monitored at 214 nm via 32 Karat software.

### Enrichment of disulfide isoforms

Redox enrichment of the IgG2-A or IgG2-B disulfide isoform was performed as previously reported^7^. The enrichment buffer for the B isoform was composed of 0.3 M Tris-HCl (pH 8.6), 5 mM L-cystamine, 0.5 mM L-cystamine, and the enrichment buffer for the A isoform had an additional 1 M guanidine hydrochloride. The samples were placed in a 2–8 °C refrigerator in the dark for 24 h, followed by dialysis against ultrapure water via Slide-A-Lyzer™ cassettes with a 10 K MWCO.

### CEX-HPLC fractionation of disulfide isoforms

A CEX-HPLC method was utilized to generate fractions enriched with IgG2 disulfide isoforms. This analysis was performed via an Agilent 1260 Infinity II system with a fraction collector. An analytical YMC BioPro IEX SF column (4.6 mm ID × 100 mm) was used to separate the IgG2 disulfide isomers. Mobile phase A consisted of 20 mM sodium acetate at pH 4.9 ± 0.05. Mobile phase B consisted of 20 mM sodium acetate and 0.5 M NaCl at pH 4.9 ± 0.05. The column was equilibrated with 19% B for 10 min, and the IgG2 disulfide isoforms were eluted via a linear gradient ranging from 19% to 52% B over 44 min. The flow rate was 0.5 ml/min, and UV absorbance at 280 nm was used for detection. The column temperature was 37 °C. Fractions were collected automatically via a fraction collector.

### Glycan profiling

N-glycans were released from 15 µg of protein and labeled with RapiFluor-MS Reagent (GlycoWorks RapiFluor-MS N-linked glycan analytical kit, Waters). The samples were analyzed on an ACQUITY-UPLC Glycan BEH amide column (130 A, 1.7 μm, 2.1 mm × 150 mm, Waters) via a fluorescence detection method. The analysis was performed via a binary gradient of 50 mM ammonium formate, pH 4.4 (A), and acetonitrile (B). The gradient conditions were as follows: the proportion of phase B decreased from 75% to 54% over a period of 35 min at a flow rate of 0.4 mL/min. This was followed by a further decrease to 0% B over a period of 1.5 min with a 3-minute isocratic hold at a flow rate of 0.2 mL/min. The proportion of phase B then increased from 0% to 75% over a period of 3.6 min at a flow rate of 0.2 mL/min. Thereafter, the proportion of phase B remained constant, but the flow rate was changed from 0.2 mL/min to 0.4 mL/min within 4.5 min and then maintained for 17.4 min. The column temperature was maintained at 60 °C throughout. Fluorescence detection was performed with the following settings: excitation/emission wavelengths of 265/425 nm and an injection volume of 2 µL.

### Surface plasmon resonance (SPR)

The binding affinity of romosozumab for sclerostin was determined via a Biacore T200 instrument (Cytiva). A goat anti-human IgG antibody (109-005-098, Jackson ImmunoResearch) was immobilized on a CM5 biosensor chip (129149603, Cytiva) via an Amine Coupling Kit (BR100050, Cytiva). Then, romosozumab (5 µg/mL) was injected at a flow rate of 10 µL/min for 30 s. A gradient concentration (0, 0.03125, 0.0625, 0.125, 0.25, 0.5, or 1 µg/mL) of human sclerostin (HST-H5245, Acro Biosystems) was then flowed over the chip surface at a flow rate of 30 µl/min for 60 s. To dissociate the analyte, running buffer (HBS-EP+, pH 7.4) was injected at a flow rate of 30 µL/min for 60 s. The data were fitted via a 1:1 model with BIAevaluation software (version 4.1) to calculate the binding constant (Ka), dissociation constant (Kd) and affinity constant (KD). The formula for calculating the affinity constant (KD) is presented as follows:

$$\:\mathrm{A}\mathrm{f}\mathrm{f}\mathrm{i}\mathrm{n}\mathrm{i}\mathrm{t}\mathrm{y}\:\mathrm{c}\mathrm{o}\mathrm{n}\mathrm{s}\mathrm{t}\mathrm{a}\mathrm{n}\mathrm{t}\:\left(\mathrm{K}\mathrm{D}\right)=\frac{\mathrm{D}\mathrm{i}\mathrm{s}\mathrm{s}\mathrm{o}\mathrm{c}\mathrm{i}\mathrm{a}\mathrm{t}\mathrm{i}\mathrm{o}\mathrm{n}\:\mathrm{c}\mathrm{o}\mathrm{n}\mathrm{s}\mathrm{t}\mathrm{a}\mathrm{n}\mathrm{t}\:\left(\mathrm{K}\mathrm{d}\right)}{\mathrm{B}\mathrm{i}\mathrm{n}\mathrm{d}\mathrm{i}\mathrm{n}\mathrm{g}\:\mathrm{c}\mathrm{o}\mathrm{n}\mathrm{s}\mathrm{t}\mathrm{a}\mathrm{n}\mathrm{t}\:\left(\mathrm{K}\mathrm{a}\right)}$$

### ELISA

The 96-well plates were coated with 500 ng/ml recombinant sclerostin at 2–8 °C overnight. 200.

A total of µL of blocking buffer (3% w/v nonfat dry milk in PBS) per well was used to block residual protein-binding sites in the wells for 1.5 h. One hundred microlitres of each diluted romosozumab solution was added to each empty well and incubated at room temperature for 2 h. The 1/50,000 diluted HRP-conjugated goat anti-human antibody (catalog no. A8667-2 mL; Sigma‒Aldrich) was then added to each well and incubated at room temperature for 1 h. Then, 100 µL of TMB was added to each well, and the plate was incubated at room temperature in the dark for 10 min, followed by the addition of 100 µL of stop solution to each well. Finally, the absorbance at 450 nm and 630 nm was detected via a plate reader (Bio-Rad Laboratories, Inc., Hercules, CA, USA). The binding activity was determined by the EC_50_. The relative potency was calculated as follows:

$$\:\mathrm{R}\mathrm{e}\mathrm{l}\mathrm{a}\mathrm{t}\mathrm{i}\mathrm{v}\mathrm{e}\:\mathrm{p}\mathrm{o}\mathrm{t}\mathrm{e}\mathrm{n}\mathrm{c}\mathrm{y}\:\left(\mathrm{\%}\right)=\frac{{EC}_{50}\:of\:RMP}{{EC}_{50}\:of\:samples}\times\:100\%$$

### Cell-based reporter gene assay

The cell-based reporter gene assay was performed as previously reported by Wei et al.^42^. Effector and target cells were trypsinized, mixed, and plated at 100 µL/well in a white-bottomed 96-well plate (Cat. No. 3610, Corning Inc., Corning, NY, USA) to achieve working densities of 2–6 × 10^3^ cells/well for effector cells and 1–3 × 10^4^ cells/well for target cells. The serially diluted antibody was preincubated with 1.25, 2.5, or 5.0 µg/mL sclerostin for 1 h at room temperature before being added immediately after cell seeding. The cells were lysed for a luciferase assay 20 h after seeding. A four-parameter model was used to analyze the corresponding relationship between relative luciferase units (RLUs) and the logarithm of sample concentrations. The relative potency was calculated as follows:

$$\:\mathrm{R}\mathrm{e}\mathrm{l}\mathrm{a}\mathrm{t}\mathrm{i}\mathrm{v}\mathrm{e}\:\mathrm{p}\mathrm{o}\mathrm{t}\mathrm{e}\mathrm{n}\mathrm{c}\mathrm{y}\:\left(\mathrm{\%}\right)=\frac{{EC}_{50}\:of\:RMP}{{EC}_{50}\:of\:samples}\times\:100\%$$

## Supplementary Information

Below is the link to the electronic supplementary material.

Supplementary Material 1

## Data Availability

The datasets generated and/or analyzed during the current study are available from the corresponding author upon reasonable request.
