# Supplementary material for: Similarity assessment of disulfide isoform profiles between the romosozumab biosimilar and the reference medicinal product
Source: Sci Rep. 2026 May 7;16:21058. doi: 10.1038/s41598-026-51578-9 (PMC13342464; doi:10.1038/s41598-026-51578-9)
Supplement: Supplementary file 1 — Supplementary Material 1 [file 41598_2026_51578_MOESM1_ESM.docx]

**Similarity assessment of the disulfide isoform profile of the romosozumab biosimilar to the reference medicinal product**

Dandan Zhao^1^, Yanling Liu^1^, Chun Wu^1^, Chunlai Cao^1^, Bohao Zhou^1^, Yongchun Lin^1^, Qiumei Liu^1^, Guanheng Li^1^, Jianrui Shi^1^, Wenyu Chen^1^, Yongjie Lai^3, *^, Jing Li^1, 2, *^

1 Zhuhai United Biopharma Co., Ltd, 399 Airport West Road, Zhuhai, Guangdong, China

2 Zhuhai United Laboratories Co., Ltd, 2428 Anji Road, Zhuhai, Guangdong, China
3 Department of Microbiology and Immunology, Zunyi Medical University (Zhuhai Campus), 368 Golden Coast Avenue, Zhuhai, Guangdong, China

* Correspondence:

Yongjie Lai

Email: laiyongjie121@163.com

Tel: 0086-756-7623304

https://orcid.org/0009-0004-9729-6836

Jing Li

Email: [lijvica@163.com](mailto:lijvica@163.com)

Tel: 0086-756-7699219
<https://orcid.org/0000-0002-7418-8101>


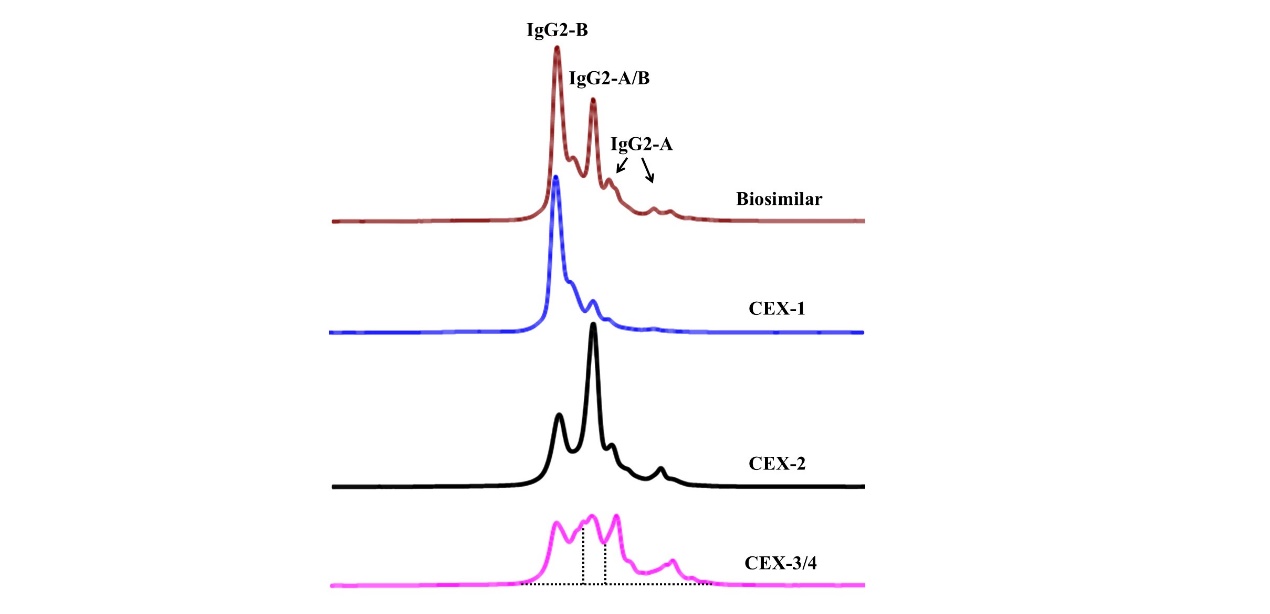


Supplementary Fig. 1. RP-HPLC analysis of CEX-HPLC fractionated CEX-1, CEX-2, and CEX-3/4 in conjunction with the original biosimilar sample.
